# Improved passive catheter tracking with positive contrast for CMR-guided cardiac catheterization using partial saturation (pSAT)

**DOI:** 10.1186/s12968-017-0368-0

**Published:** 2017-08-15

**Authors:** Mari Nieves Velasco Forte, Kuberan Pushparajah, Tobias Schaeffter, Israel Valverde Perez, Kawal Rhode, Bram Ruijsink, Mazen Alhrishy, Nicholas Byrne, Amedeo Chiribiri, Tevfik Ismail, Tarique Hussain, Reza Razavi, Sébastien Roujol

**Affiliations:** 1 Division of Imaging Sciences and Biomedical Engineering, King’s College London, St Thomas’ Hospital, 3rd Floor Lambeth Wing, Westminster Bridge Road, London, SE1 7EH UK; 2 Department of Congenital Heart Disease, Evelina London Children’s Hospital, Guy’s and St Thomas’ NHS Foundation Trust, London, UK; 3 Cardiovascular Pathology Unit, Institute of Biomedicine of Seville, IBIS, Virgen del Rocio University Hospital/CSIC/University of Seville, Seville, Spain; 4 Department of Medical Physics, Guy’s and St. Thomas’ NHS Foundation Trust, London, UK; 5 Dept. of Pediatrics, University of Texas Southwestern Medical Center, 1935 Medical District Drive, Dallas, USA

**Keywords:** Interventional CMR, Congenital heart disease, Cardiac catheterization, Device tracking

## Abstract

**Background:**

Cardiac catheterization is a common procedure in patients with congenital heart disease (CHD). Although cardiovascular magnetic resonance imaging (CMR) represents a promising alternative approach to fluoroscopy guidance, simultaneous high contrast visualization of catheter, soft tissue and the blood pool remains challenging. In this study, a novel passive tracking technique is proposed for enhanced positive contrast visualization of gadolinium-filled balloon catheters using partial saturation (pSAT) magnetization preparation.

**Methods:**

The proposed pSAT sequence uses a single shot acquisition with balanced steady-state free precession (bSSFP) readout preceded by a partial saturation pre-pulse. This technique was initially evaluated in five healthy subjects. The pSAT sequence was compared to conventional bSSFP images acquired with (SAT) and without (Non-SAT) saturation pre-pulse. Signal-to-noise ratio (SNR) of the catheter balloon, blood and myocardium and the corresponding contrast-to-noise ratio (CNR) are reported. Subjective assessment of image suitability for CMR-guidance and ideal pSAT angle was performed by three cardiologists. The feasibility of the pSAT sequence is demonstrated in two adult patients undergoing CMR-guided cardiac catheterization.

**Results:**

The proposed pSAT approach provided better catheter balloon/blood contrast and catheter balloon/myocardium contrast than conventional Non-SAT sequences. It also resulted in better blood and myocardium SNR than SAT sequences. When averaged over all volunteers, images acquired with a pSAT angle of 20° to 40° enabled simultaneous visualization of the catheter balloon and the cardiovascular anatomy (blood and myocardium) and were found suitable for CMR-guidance in >93% of cases. The pSAT sequence was successfully used in two patients undergoing CMR-guided diagnostic cardiac catheterization.

**Conclusions:**

The proposed pSAT sequence offers real-time, simultaneous, enhanced contrast visualization of the catheter balloon, soft tissues and blood. This technique provides improved passive tracking capabilities during CMR-guided catheterization in patients.

**Electronic supplementary material:**

The online version of this article (doi:10.1186/s12968-017-0368-0) contains supplementary material, which is available to authorized users.

## Background

The incidence of congenital heart disease (CHD) in Europe has recently been estimated at 8.2 per 1000 live births [1]. Cardiac catheterization is a common diagnostic and interventional procedure in patients with CHD [2, 3] which has classically been performed under fluoroscopic guidance.

Cardiovascular magnetic resonance imaging (CMR)-guidance of cardiac catheterization procedures is a promising alternative to fluoroscopy as it avoids ionising radiation and provides improved real-time anatomical visualization of the cardiovascular structures [4–6]. This approach has been evaluated in a large number of pre-clinical studies [7–20]. However, clinical cardiac catheterization using CMR has been established in only a few centres [4, 21–27], mainly for diagnostic purposes. Very few interventional procedures have been carried out under CMR guidance [26, 28, 29]. Clinical translation has been hindered by a lack of suitable CMR-compatible catheters and guide-wires and also by the limited capabilities of current visualization techniques.

Current approaches for real-time catheter visualization during CMR-guided catheterization include active and passive tracking approaches [30]. Active tracking has been used in CMR-guided electrophysiology studies [31–34]. However, there are no active catheters or guide-wires in clinical use for congenital/structural heart disease with only pre-clinical experience in transcatheter aortic valve implantation and mitral valve interventions [16, 35].

Non-metallic catheters and guide-wires have been used for passive tracking in CMR-guided cardiac catheterization [24–26]. Balloon wedge catheters can be filled with gadolinium for positive contrast or CO_2_ for negative contrast (signal void) [26]. In order to improve the visualization of gadolinium-filled balloon catheters in real-time CMR, the interactive application of saturation pre-pulses during the intervention has been proposed [24]. Application of the saturation pulse maximizes the positive contrast between the gadolinium-filled balloon and the anatomy, for which visualization is reduced. Conversely, images acquired without saturation pre-pulses enable excellent anatomical visualization (blood and myocardium) but provide poor catheter visualization due to reduced contrast. Black blood preparation using flow sensitive gradients has been proposed to preserve soft tissue signal and simultaneously visualize the catheter balloon [36]. However, this technique also suppresses the blood signal and may result in suboptimal visualization of thinned walled anatomical structures such as the atriums and great vessels.

In this study, we developed and evaluated a novel positive contrast-based passive tracking sequence using partial saturation (pSAT) magnetization preparation, to provide simultaneous enhanced contrast visualization of both cardiovascular anatomy and gadolinium-filled balloon catheters. This technique was initially evaluated in healthy subjects and subsequently applied to CMR-guided catheterization in two patients with CHD.

## Methods

All imaging was performed on a Philips XMR system (Achieva, Philips, Best, Netherlands), which consists of a 1.5 T CMR-scanner and a BV Pulsera cardiac X-Ray unit. A 5-channel cardiac phased array receiver coil was used for all imaging experiments. All experiments were conducted using a balloon wedge catheter (Arrow Intl., Inc., Reading, Pennsylvania, USA) filled with a dilution of 1% of gadolinium (Dotarem®, Guerbet LLC, Bloomington, Indiana, USA) mixed with a physiological 0.9% sodium chloride solution. The study was approved by the National Research Ethics Service, with written informed consent obtained from all participants.

### Passive tracking sequence using a partial saturation pre-pulse

A novel passive tracking sequence is proposed using real-time single-shot acquisition with bSSFP readout (TR/TE = 2.65 ms/1.3 ms, flip angle = 60°, FOV = 350 × 300 mm^2^, voxel size = 2.2 × 2.5 mm^2^, slice thickness = 10 mm, bandwidth = 1250 Hz/pixel, SENSE factor = 2, partial k-space acquisition in the phase encoding direction = 65%, number of phase encoding lines = 40, acquisition time = 106 ms, linear ordering, extra “dead time” of ~64 ms after each readout due to SAR constraints (2 W/kg) required for paediatric use). Each image is acquired immediately after a saturation pre-pulse with a reduced saturation angle to achieve partial saturation. The time between the pSAT pulse and the readout of the k-space center line (referred to as saturation delay time) was 41 ms. A non-selective saturation pulse provided and optimized by the manufacturer for regional saturation technique (REST) was used with the following parameters: bandwidth = 3966 Hz and duration = 2.1 ms with modulation of pulse power to reach the desired pSAT angle. Slight modifications of the pulse (duration = 2.3 ms and bandwidth = 3555 Hz) were employed for a pSAT angle of 90° to satisfy B1/SAR limits. This sequence has a temporal resolution of ~5 Hz. The sequence takes approximately 7 images (1.5 s) to reach steady-state resulting in a progressive improvement in visibility thereafter. Unless stated otherwise, all experiments in this study used the sequence parameters described in this section. The use of the pSAT sequence with a pSAT angle of 0° (i.e. saturation pulse turned off) and 90° are hereafter referred to as Non-SAT and SAT sequences, respectively.

### In-vivo evaluation in healthy subjects

The pSAT sequence was evaluated in five healthy subjects (33 ± 3 year-old, 4 male). A passively tracked CMR guided cardiac catheterization was approximated by positioning a gadolinium-filled balloon catheter on the chest of each volunteer. A large syringe containing the same concentration of gadolinium was also placed on the chest to obtain additional “reference” signal free from partial volume averaging. The pSAT sequence was run using 10 different pSAT angles (0° to 90°, in steps of 10°), each acquired with 5 different slice thicknesses ([5, 7, 10, 15, 20] mm using a framerate of 5 Hz and saturation time delay of 41 ms), and 3 different framerates ([5, 3.5, 2.5] Hz using a slice thickness of 10 mm and saturation delay times of [41,200,300] ms, respectively). This resulted in a total of 70 pSAT acquisitions per subject.

Catheter balloon signal-to-noise ratio (SNR), blood SNR, myocardium SNR, catheter balloon/blood contrast-to-noise ratio (CNR), catheter balloon/myocardium CNR, and blood/myocardium CNR were measured in the last image of each acquisition. Myocardial and blood signal were measured over a region of interest manually drawn within the septum and the right ventricle cavity, respectively. SNR and CNR were evaluated using the NEMA approach [37]. The noise, n, was approximated from the difference between two images acquired with the same pSAT sequence with identical parameters, as follows: $n=SD/\sqrt{2}$, where SD is the standard deviation of the signal over a region of interest manually drawn over the right ventricle of the difference image.

Qualitative evaluation of the pSAT sequence was then performed by three cardiologists with more than 5 years of experience in cross-sectional imaging and CMR catheterization. This analysis was limited to the pSAT sequence acquisitions (pSAT angle from 0° to 90°) using a 10 mm slice thickness and a framerate of 5 Hz. Subjective assessment of the contrast between catheter balloon and blood and quality of visualization of cardiovascular structures were scored using a 5-point scale (see Tables 1 and 2 for description of the scoring system) [38]. Experts were able to window the level of image contrast as they wished and were asked to score the 20th image of each acquisition. The mean of the experts’ scores was then calculated. Subjective assessment of image suitability for CMR-guidance and ideal pSAT angle were also evaluated for each healthy volunteer. Each expert scored the suitability of each acquisition for CMR-guidance as: 0 (insufficient contrast between balloon and cardiovascular structures or insufficient blood/myocardium SNR to carry out the procedure) or 1 (sufficient contrast between balloon and cardiovascular structures and sufficient blood/myocardium SNR). Furthermore, each expert was asked to determine the ideal pSAT acquisition (i.e. pSAT angle) for each volunteer.

**Table 1:** Image quality scoring system for visualization of cardiovascular structures

Score	Description
1	Poor. Unable to differentiate cardiovascular structures
2	Low. Cardiovascular structures can be slightly intuited.
3	Intermediate. Cardiovascular structures can be delineated.
4	Good. Good definition of cardiovascular structures.
5	Excellent. Optimal cardiovascular structures delineation.

**Table 2:** Image quality scoring system for contrast between blood pool and balloon of the wedge catheter

Score	Description
1	Poor. Impossible to differentiate balloon and blood pool.
2	Low. Differentiation of blood and balloon difficult to perform.
3	Intermediate. Adequate signal disparity.
4	Good. Differentiation of blood and balloon easily performed.
5	Excellent. Excellent contrast between blood and balloon

### In-vivo CMR-guided catheterization in patients

Two patients (39 and 15 year old females) in whom XMR cardiac catheterization was clinically indicated for pulmonary vascular resistance (PVR) assessment prior to consideration of further management were included. These patients had uncorrected atrioventricular septal defect and hypoplastic left heart syndrome post-Fontan completion, respectively. Both procedures were performed under general anaesthesia. A 6Fr sheath and a 20G arterial Laedercath (Vygon, Swindon, UK) were placed in the femoral vein and artery respectively for each patient. A 6Fr balloon wedge catheter (Arrow Intl., Inc., Reading, Pennsylvania, USA) was used throughout the CMR-guided procedure [25]. In the first patient, the pSAT sequence was run several times with different pSAT angles (10° to 60° in steps of 10°). CMR-guided catheterization was performed using the pSAT sequence with a pSAT angle of 30° and 40° for the first and second patient, respectively.

After the procedure, qualitative analysis of the same metrics used for the healthy volunteer study was performed using the preliminary datasets acquired with different pSAT angles in the first patient.

## Results

### In-vivo evaluation in healthy subjects

**Fig. 1:**
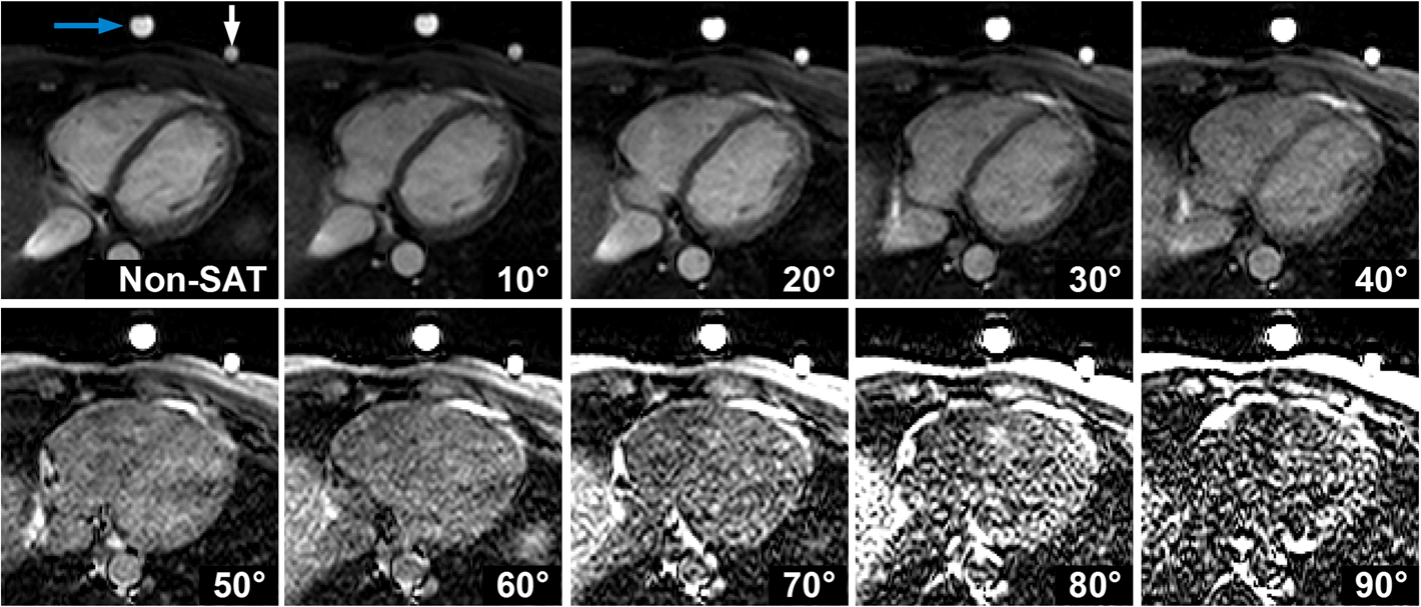
Images acquired with the pSAT sequence in one healthy volunteer using different pSAT angles (*Blue arrow* = syringe, *white arrow* = balloon wedge catheter). Image contrast was adjusted for each image to a window with minimum of 0 and maximum equal to twice the average right ventricular blood signal. Intermediate pSAT angles of 20°-40° enabled simultaneous visualization of balloon, blood and myocardium

Figure 1 shows real-time images acquired with the pSAT sequence in one healthy volunteer. Increased of pSAT angle was associated with higher balloon/blood contrast but reduced ability to visualize cardiovascular structures. Low pSAT angles (<20°) resulted in poor balloon/blood contrast, while larger pSAT angle (>40°) led to substantial reduction of blood and myocardium signal. Intermediate pSAT angles of 20°-40° enabled simultaneous visualization of balloon, blood and myocardium.

**Fig. 2:**
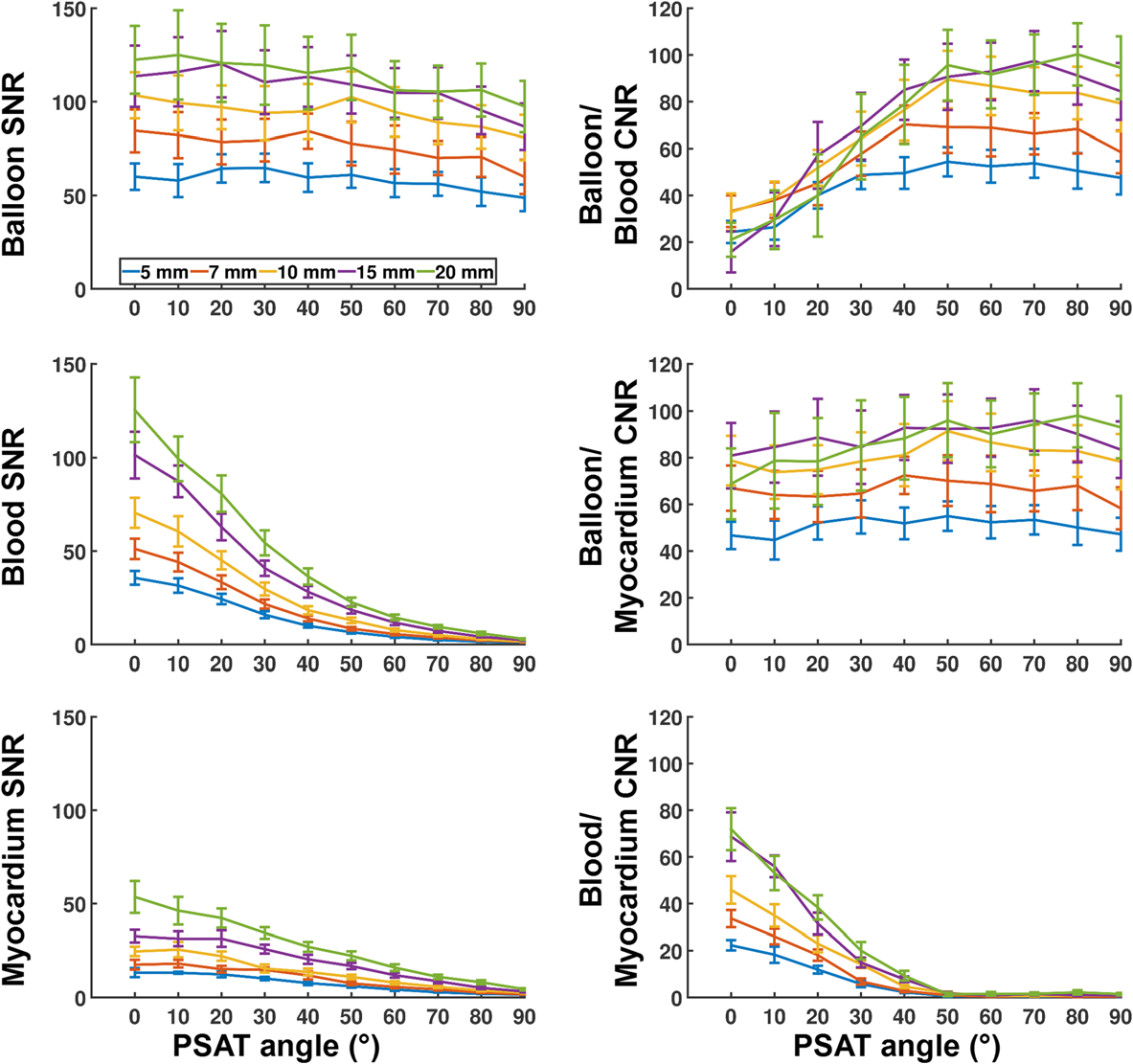
Influence of slice thickness and pSAT angle on the proposed pSAT sequence measured in healthy subjects. Data are shown as average and standard deviation over all subjects. Low pSAT angles (<20°) resulted in reduced balloon/blood CNR, while high pSAT angles (>40°) led to reduced blood and myocardium SNR. In the pSAT range of interest (20° to 40°), larger slice thicknesses resulted in higher SNRs and CNRs

The influence of the pSAT angle and slice thickness on the pSAT sequence measured in healthy subjects is shown in Fig. 2. To describe the influence of the pSAT angle on the pSAT sequence, the data from Fig. 2 were first averaged over all slice thicknesses and are reported as average SNR/CNR variation ± standard deviation (over all subjects) between the Non-SAT sequence and the pSAT sequence using each of the following pSAT angles: 20°, 40°, 90°. Balloon SNR reduced by 1 ± 6%, 4 ± 5%, 23 ± 10%, respectively. Blood SNR reduced by 36 ± 2%, 72 ± 1%, 97 ± 1%, respectively. Myocardium SNR reduction was 12 ± 4%, 42 ± 7%, 90 ± 2%, respectively. Balloon/blood CNR increased by 89 ± 31%, 207 ± 77%, 212 ± 68%, respectively. Low balloon/myocardium CNR variation was observed (<15% in all cases). Blood/myocardium CNR reduced by 49 ± 9%, 88 ± 1%, 98 ± 1%, respectively, demonstrating that most of this contrast vanishes with pSAT angle >40°.

To describe the influence of slice thickness on the pSAT sequence, the data from Fig. 2 were first averaged over all subjects and are reported as average SNR/CNR variations ± standard deviation (over all pSAT angles) between the smaller slice thickness (5 mm) and each of the larger slice thicknesses: 7 mm, 10 mm, 15 mm, 20 mm. Balloon SNR increased by 30 ± 8%, 62 ± 9%, 84 ± 8%, 96 ± 10%, respectively. Blood SNR increased by 38 ± 10%, 86 ± 18%, 169 ± 30%, 242 ± 40%, respectively. Myocardium SNR increased by 34 ± 14%, 82 ± 13%, 160 ± 26%, 262 ± 36%, respectively. In average over all pSAT angles >20°, Balloon/blood CNR increased by 29 ± 8%, 58 ± 12%, 71 ± 13%, 74 ± 22%, respectively. Balloon/myocardium CNR increased by 30 ± 9%, 60 ± 9%, 75 ± 9%, 72 ± 17%, respectively. In average over all pSAT angles <50°, Blood/myocardium CNR increased by 38 ± 15%, 107 ± 22%, 195 ± 33%, 237 ± 43%, respectively.

**Fig. 3:**
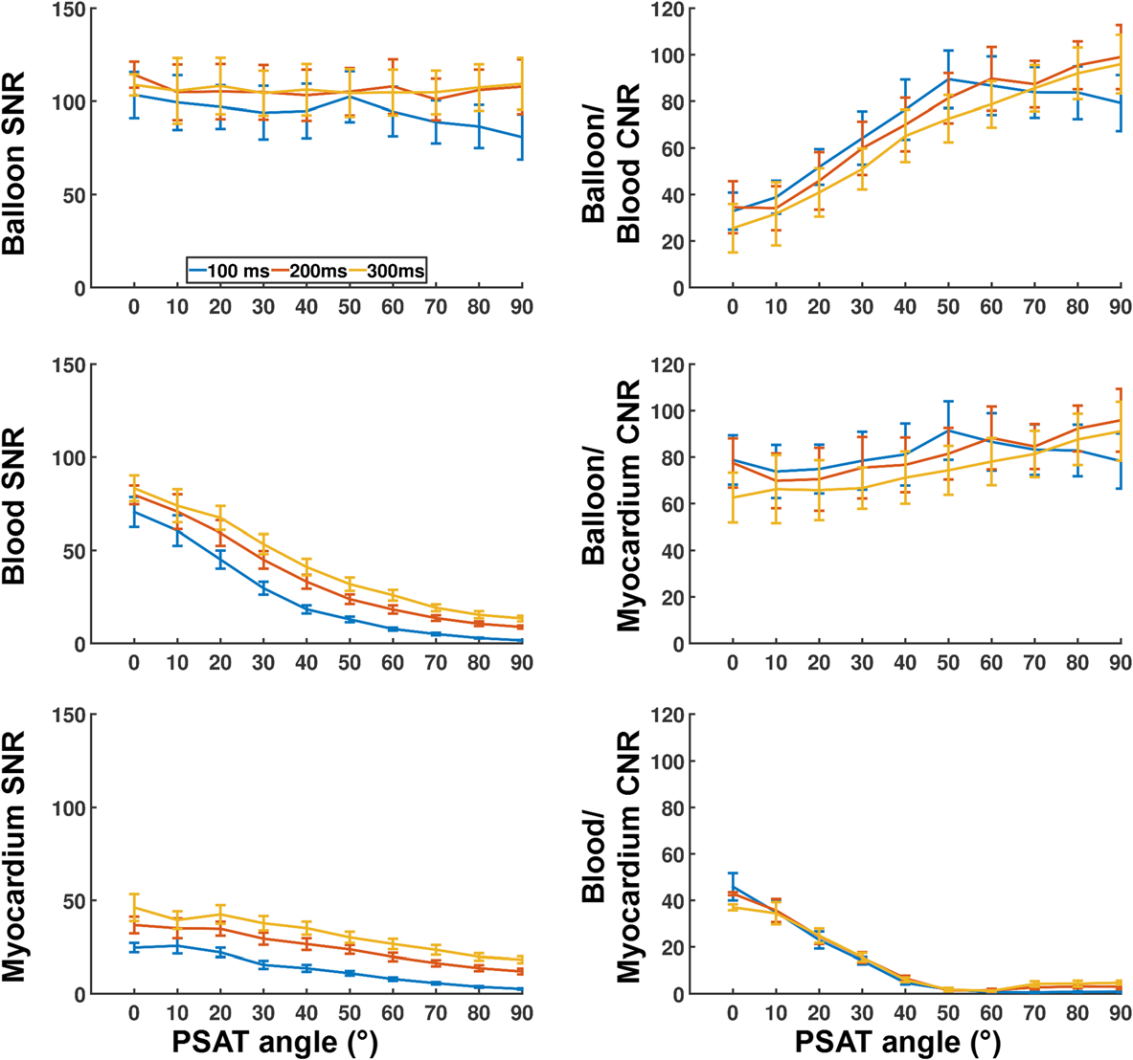
Influence of framerate and pSAT angle on the proposed pSAT sequence measured in healthy subjects. Data are shown as average and standard deviation over all subjects. Low pSAT angles (<20°) resulted in reduced balloon/blood CNR, while high pSAT angles (>40°) led to reduced blood and myocardium SNR. In this pSAT range of interest (20° to 40°), lower framerate resulted in higher balloon, blood, and myocardium SNR, but reduced balloon/blood CNR and balloon/myocardium CNR

The influence of the pSAT angle and framerate on the pSAT sequence measured in healthy subjects is shown in Fig. 3. To describe the influence of framerate on the pSAT sequence, the data from Fig. 3 were first averaged over all subjects and are reported as average absolute SNR/CNR variations ± standard deviation (over all pSAT angles) between a framerate of 5 Hz and each of the two following framerates: 3.3 Hz and 2.5 Hz. Balloon SNR increased by 12 ± 7 and 12 ± 8, respectively. Blood SNR increased by 11 ± 3 and 17 ± 5, respectively. Myocardium SNR increased by 12 ± 2 and 18 ± 3, respectively. In average over all pSAT angles <60°, balloon/blood CNR reduced by 4 ± 3 and 10 ± 3, respectively, and balloon/myocardium CNR reduced by 3 ± 1 and 11 ± 3, respectively. All framerates provided similar blood/myocardium CNR (all variations < 1).

**Fig. 4:**
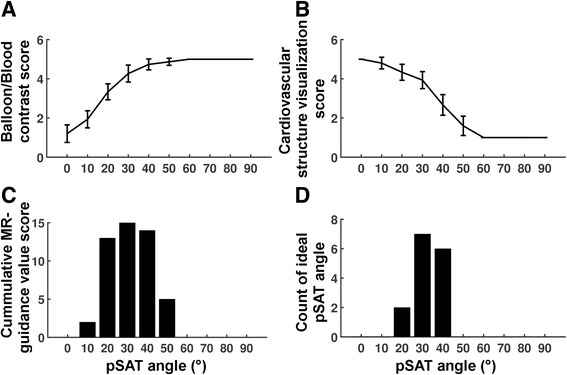
Subjective assessment of the pSAT sequence in healthy subjects. Catheter balloon/blood contrast (**a**), cardiovascular structure visualization (**b**), image suitability score for CMR-guidance (**c**), and ideal pSAT angle (**d**) are shown. In (**a**) and (**b**), scores were first averaged over all experts and are shown in average ± standard deviation over all subjects. A pSAT angle range of 20°-40° resulted in balloon/blood contrast score > 2, cardiovascular structure visualization score > 2 and image suitability for CMR-guidance in >93% of cases. The average ideal pSAT angle was 33 ± 7°

The subjective analysis of the healthy subject study is summarized in Fig. 4. Low pSAT angles (<20°) resulted in low balloon/blood contrast (scores <2). High pSAT angles (>40°) led to low quality visualization of cardiovascular structures (scores <2). A pSAT angle range of 20°-40° resulted in an average score for both visualization of cardiovascular structures and balloon/blood contrast >2 and was considered suitable for CMR-guidance in >93% of cases. Optimal pSAT angles were subjectively assessed to be 20–40°.

### In-vivo CMR-guided catheterization in patients

**Fig. 5:**
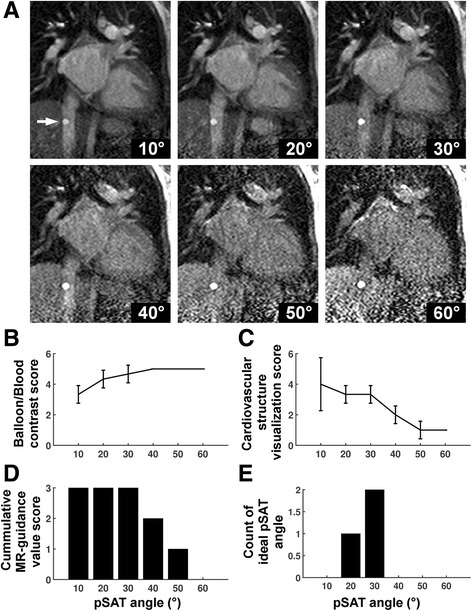
In-vivo analysis of the pSAT sequence in one patient undergoing CMR-guided catheterization. The catheter was positioned at the proximal inferior vena cava (*arrow*). **a** Images acquired using the pSAT sequence with different pSAT angles. Image contrast was adjusted for each image to a window with minimum of 0 and maximum equal to twice the average right ventricular blood signal. **b**-**e** Qualitative assessment of the pSAT sequence including catheter balloon/blood contrast (**b**), cardiovascular structure visualisation score (**c**), image suitability score for CMR-guidance (**d**), and ideal pSAT angle (**e**). A pSAT angle range of 10°-30° resulted in balloon/blood score > 2, cardiovascular structure visualization score > 2 and image suitability for CMR-guidance in 100% of cases. The average ideal pSAT angle was 27 ± 6°

**Fig. 6:**
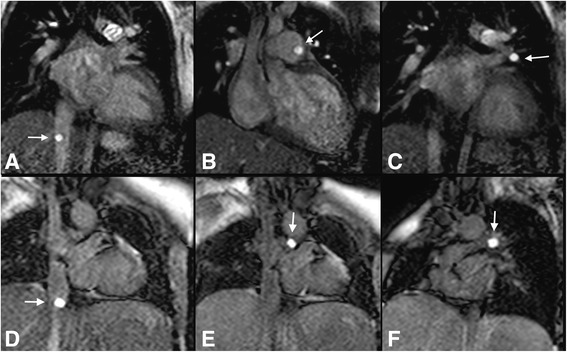
Real-time images acquired in two patients during CMR-guided catheterization using the pSAT sequence with a pSAT angle of 30° (patient #1, **a**-**c**) and 40° (patient #2, **d**-**f**). Note balloon of the wedge catheter (see *white arrow*) at the inferior vena cava (**a**), main pulmonary artery (**b**) and left pulmonary wedge (**c**) in patient 1; and in the IVC (**d**) and LPA (**e**, **f**) in patient #2. Simultaneous visualization of catheter balloon and blood/heart structures was achieved with enhanced contrast and SNR using the proposed sequence

Figure 5a and 6 show real-time images obtained with the proposed pSAT sequence in patients with CHD. Figure 5b-e shows qualitative results obtained in the first patient. A pSAT angle range of 10°-30° resulted in balloon/blood contrast score > 2, quality of cardiovascular structure visualization score > 2 and image suitability for MR-guidance in 100% of cases. The ideal pSAT angle ranged from 20 to 30°. In the first patient, the catheter was easily guided from the IVC to the right atrium, right ventricle, main pulmonary artery and pulmonary branches; in the second patient, the balloon was guided from the IVC into the pulmonary arteries without difficulty (see Fig. 6 and Additional file 1: Video S1). The balloon and the cardiovascular structures were well visualized throughout each procedure. The first procedure was performed solely under CMR guidance. Support with x-ray was needed for the second patient (post-Fontan completion) to access the intracardiac anatomy across the fenestration from the lateral tunnel of the Fontan pathway using a wire.

## Discussion

In this study, we developed and evaluated a novel passive tracking sequence using a pSAT pre-pulse. This technique provides simultaneous visualization of the tip of the catheter and the patient’s anatomy with higher contrast than conventional Non-SAT sequences and better SNR than conventional SAT sequences. The clinical feasibility of CMR-guided catheterization using the proposed pSAT sequence was successfully demonstrated.

Good agreement was found between the healthy subject study and the patient study. The choice of the ideal pSAT angle consists in finding the best compromise between sufficient blood and myocardium SNR (which gradually deteriorates with increasing pSAT angles) and sufficient catheter balloon/blood CNR (which gradually improves with increasing pSAT angles of up to 50°). Overall, a pSAT angle of 20° to 40° was found to provide a good compromise between visualization of the cardiovascular anatomy (blood and myocardium) and catheter balloon/blood contrast for all employed slice thicknesses and framerates. In this pSAT angle range, larger slice thicknesses increased all measured SNRs and CNRs, while slower framerates increased all measured SNRs but decreased balloon/blood CNR and balloon/myocardium CNR. Therefore, this approach may enable the use of larger slice thicknesses and reduce the problem of “out-of-plane” catheters.

Previous clinical CMR catheterization studies have used other types of saturation pulses such as interactive on/off saturation pulses [24], or flow sensitive preparation pulses for blood signal suppression [36]. The proposed approach has several advantages. It provides simultaneous visualization of soft tissue, blood and catheter balloon. Furthermore, this approach is easy to implement and to use as it only requires the adjustment of the saturation angle, which can often be done from most standard scanner consoles.

A frame-rate of 5 images/s was used in this study. Higher frame-rates have been reported [26] [4]. Although the employed frame-rate was found suitable for procedure guidance, a higher frame-rate could provide improved navigation but was not investigated in the current study. Advanced acceleration techniques such as temporal sensitivity encoding (TSENSE) [39] or temporal Generalized Autocalibrating Partially Parallel Acquisitions (TGRAPPA) [40] potentially combined with computationally efficient reconstruction [41] could be used to further improve the frame-rate. Further studies are required to investigate the benefit of higher framerate using the proposed pSAT sequence.

Our study has several limitations. Firstly, the use of a catheter positioned on the healthy subjects' chest may have introduced some bias in terms of quantitative and qualitative analysis, when compared to real-life scenario where the catheter is navigated through the cardiovascular system. However, these findings were similar with in-vivo studies in two patients. Second, the use of low framerates with short time delays was not investigated in this study. Finally, we have evaluated the clinical feasibility of the pSAT approach in two patients. Larger clinical studies will be needed to establish its clinical value over existing techniques.

## Conclusion

The proposed pSAT sequence offers real-time simultaneous enhanced contrast visualization of the catheter balloon, soft tissues and blood. This technique provides improved passive tracking capabilities during CMR-guided catheterization in patients.

## Additional file

Additional file 1:
**Video S1.** CMR-guided catheterization using the pSAT sequence in the second patient. The balloon of the wedge catheter is seen in the inferior vena cava, lateral tunnel and entering the left pulmonary artery. (MP4 8665 kb)

## Abbreviations

bSSFP: Balanced steady state free precession
CHD: Congenital heart disease
CMR: Cardiovascular magnetic resonance
CNR: Contrast-to-noise ratio
FOV: Field of view
MR: Magnetic resonance
NEMA: National Electrical Manufacturers Association
Non-SAT: Without Saturation
pSAT: Partial saturation
PVR: Pulmonary vascular resistance
SAT: Saturation
SNR: Signal-to-noise ratio
TE: Echo time
TR: Repetition time
XMR: X-ray and MRI
